# Interface-induced spin Hall magnetoresistance enhancement in Pt-based tri-layer structure

**DOI:** 10.1038/s41598-017-18369-9

**Published:** 2018-01-08

**Authors:** Shun-Yu Huang, Hong-Lin Li, Cheong-Wei Chong, Yu-Ying Chang, Min-Kai Lee, Jung-Chun-Andrew Huang

**Affiliations:** 1 Department of Physics, National Cheng Kung University, Tainan, 70101 Taiwan; 2 Institute of Physics of the ASCR, v. v. i., Cukrovarnicka 10, 16200 Praha 6, Czech Republic; 3 Faculty of Biomedical Engineering, Czech Technical University, nam. Sitna 3105, 27201 Kladno, Czech Republic; 4 Instrument Development Center, National Cheng Kung University, Tainan, 70101 Taiwan; 5 Advanced Optoelectronic Technology Center (AOTC), National Cheng Kung University, Tainan, 70101 Taiwan; 6 Taiwan Consortium of Emergent Crystalline Materials, Ministry of Science and Technology, Taipei, 106 Taiwan

## Abstract

In this study, we integrated bilayer structure of covered Pt on nickel zinc ferrite (NZFO) and CoFe/Pt/NZFO tri-layer structure by pulsed laser deposition system for a spin Hall magnetoresistance (SMR) study. In the bilayer structure, the angular-dependent magnetoresistance (MR) results indicate that Pt/NZFO has a well-defined SMR behavior. Moreover, the spin Hall angle and the spin diffusion length, which were 0.0648 and 1.31 nm, respectively, can be fitted by changing the Pt thickness in the longitudinal SMR function. Particularly, the MR ratio of the bilayer structure (Pt/NZFO) has the highest changing ratio (about 0.135%), compared to the prototype structure Pt/Y_3_Fe_5_O_12_ (YIG) because the NZFO has higher magnetization. Meanwhile, the tri-layer samples (CoFe/Pt/NZFO) indicate that the MR behavior is related with CoFe thickness as revealed in angular-dependent MR measurement. Additionally, comparison between the tri-layer structure with Pt/NZFO and CoFe/Pt bilayer systems suggests that the SMR ratio can be enhanced by more than 70%, indicating that additional spin current should be injected into Pt layer.

## Introduction

Spin-orbit coupling (SOC)^1,2^ is one of the most important phenomena in condensed matter physics, providing a mechanism to couple charge and spin of electrons, and bring several fields such like quantum spin Hall effect^3–5^ and Topological insulators^6–9^. Particularly, the most noticeable example for SOC-related phenomena is the spin Hall effect (SHE)^10–15^, which has been widely applied in spintronics since its discovery. The SHE describes the changing process of charge current and pure spin current without ferromagnetic material. In heavy metals, owing to spin-orbit interaction, the charge current can be converted into spin current in perpendicular direction. On the other hand, the inverse SHE (ISHE) explains that pure spin current can be changed back into charge current in perpendicular direction. Nowadays, several research fields, such as spin pumping^16,17^, spin transfer torque^18^, and spin Seebeck effect^19,20^, are focusing on the SHE and the ISHE.

**Figure 1:**
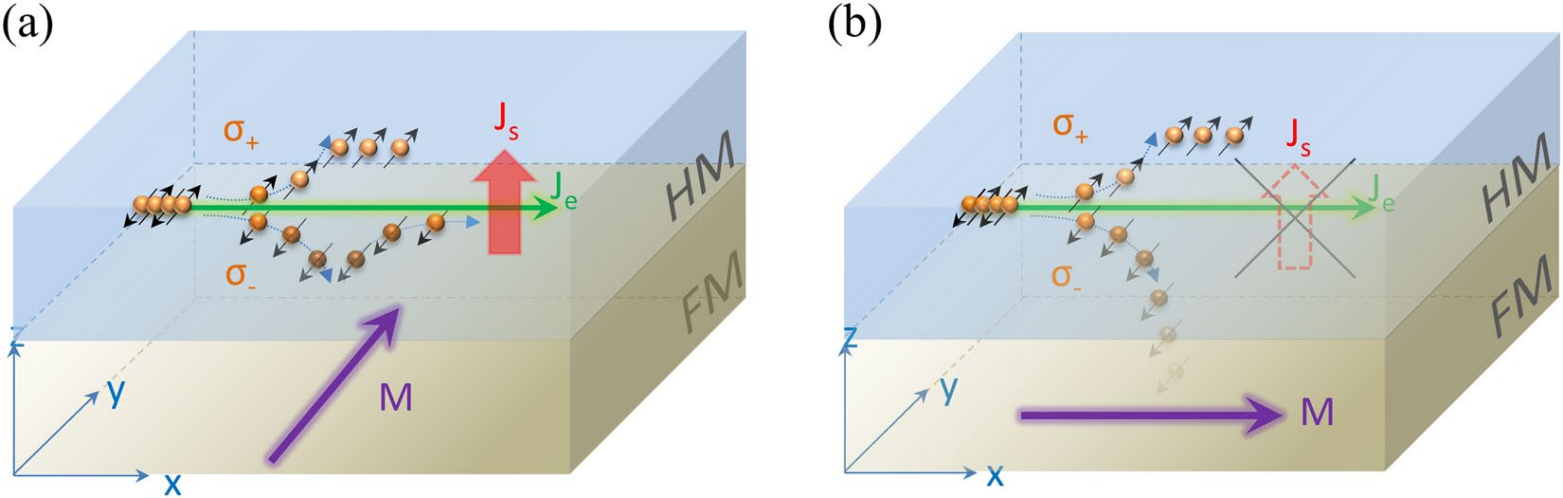
Illustrates schematic the diagrams of the SMR mechanism. (**a**) and (**b**) show a charge current (green arrow) drive into a nonmagnetic heavy metal layer (blue layer), and the spin accumulates in the top and bottom surface due to the SHE. The different magnetic moment direction (purple arrow) of the ferromagnetic layer (gray layer) can produce (**a**) reflection and (**b**) absorption of spin current at the interface, introducing low and high resistance state, respectively.

More recently, a new magnetoresistance phenomenon has been discovered, the so-called spin Hall magnetoresistance (SMR)^21,22^, which is connected with the SHE and ISHE. The SMR behavior occurs when two different materials are merged together, usually a ferromagnetic material (FM) and a paramagnetic heavy metal (HM). Figure 1(a),(b) illustrate the spin directions (black arrow) parallel and perpendicular to the magnetization of FM (purple arrow), respectively. The spin current (generated by the SHE) is reflected or absorbed by the FM at the HM/FM interfaces. Two different resistance states are then observed, as shown in Fig. 1(a),(b). Compared to other spintronic devices, SMR studies are the simplest method to obtain spin current parameters (such as the spin Hall angle or spin diffusion length) and to characterize materials^23–26^. In the early stages of the SMR research, ferromagnetic insulators (FMIs) such as YIG and CoFe_2_O_4_ (CFO) had been chosen as the magnetic layer^27–30^ because this arrangement can ensure that all currents flow only in the HM layer. Furthermore, these studies also confirm the origin of SMR and provide plentiful results in different materials. However, the application of the SMR effect has been quite difficult owing to low MR change ratios, which are only about 10^−2^ (%) to 10^−3^ (%), in HM/FMI bilayer structures. Most recently, Junyeon Kim *et al*.^31^ used ferromagnetic metal CoFeB to replace the FMI in the bilayer structure. Their results show that the SMR ratio of HM/FM bilayer structure is one order of magnitude higher than the HM/FMI bilayer^32–34^. These results have highly increased the application possibility of the SMR-related effect in spintronics. However, the main studies of the SMR behavior focus on the bilayer structure, particularly in the FMI/HM structure, while the SMR behavior itself is generated from the interface of the FM layer and HM. In general, increasing the number of interfaces may increase the total SMR ratios. However, very few studies^35^ report on the SMR behavior of tri-layer or multilayer structures. Therefore, tri-layer structure behaviors are an essential topic of research for SMR-related studies.

In this study, we systemically investigate the SMR behaviors with bilayer and trilayer structures. The work is organized as follows: first, we grew Ni_0.3_Zn_0.7_Fe_2_O_4_ (NZFO) on C-sapphire and identified their structure and magnetism behavior—NZFO is one of the ideal candidate for SMR studies due to its great insulation and high magnetization; after that, we grew Pt on NZFO because Pt is the most widely used material for generating SHE and ISHE due to its high spin orbit interaction. The field and angular-dependent MR results can help us to obtain the spin Hall angle and spin diffusion length by fitting the longitudinal SMR function in FMI/HM system. Furthermore, we grow another ferromagnetic layer CoFe on Pt/NZFO to build a tri-layer structure, which we compared with the Pt/NZFO and CoFe/Pt bilayer structure. The comparison results show that the SMR ratio of the tri-layer structure is higher than the Pt/NZFO and CoFe/Pt bilayer structures and is increased by more than 70%.

## Results and Discussion

**Figure 2:**
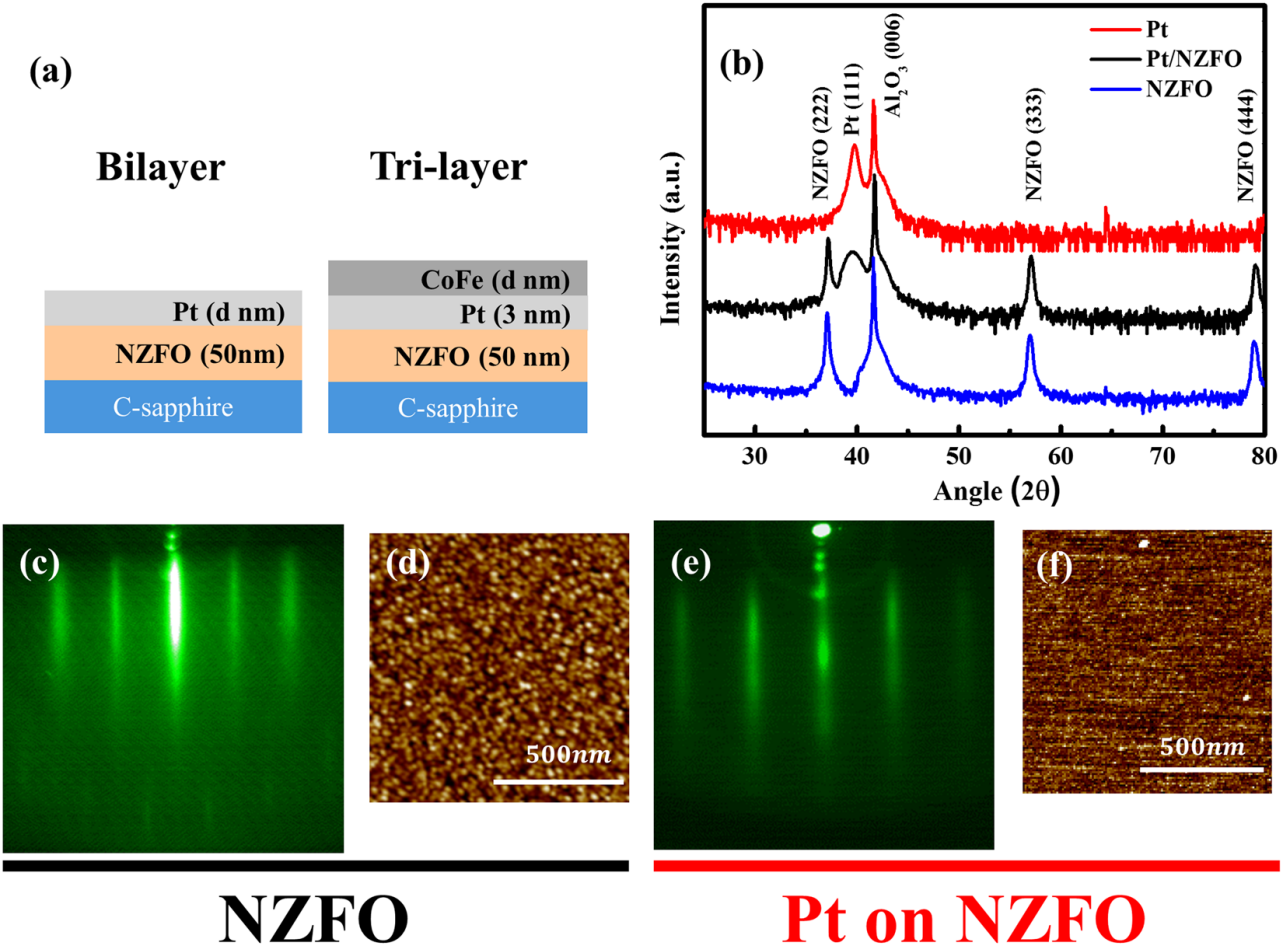
(**a**) Schematic diagrams of the bilayer and tri-layer structure. (**b**) X-ray diffraction patterns of Pt, NZFO and Pt growth on NZFO. (**c**), (**d**), (**e**), (**f**) RHEED patterns and AFM images of NZFO and Pt growth on NZFO.

XRD patterns of NZFO and Pt illustrated a clean (111) orientation without any second phase, and the pattern of Pt/NZFO bilayer structure revealed that the sample had ideal layer structure condition, as shown in Fig. 2(b). Meanwhile, Fig. 2(c),(e) show the RHEED patterns of single NZFO layer and Pt growth on NZFO layer, respectively. The patterns indicate that the NZFO and Pt growth on NZFO have smooth and ideal surface condition, which is also confirmed by the AFM result, and the surface roughness is 0.4 nm and 0.2 nm, as shown in Fig. 2(d),(f), respectively. The structure analysis results indicate that the PLD growth bilayer samples ensure high quality structure with smooth surface and appropriate structure for the SMR behaviors research.

**Figure 3:**
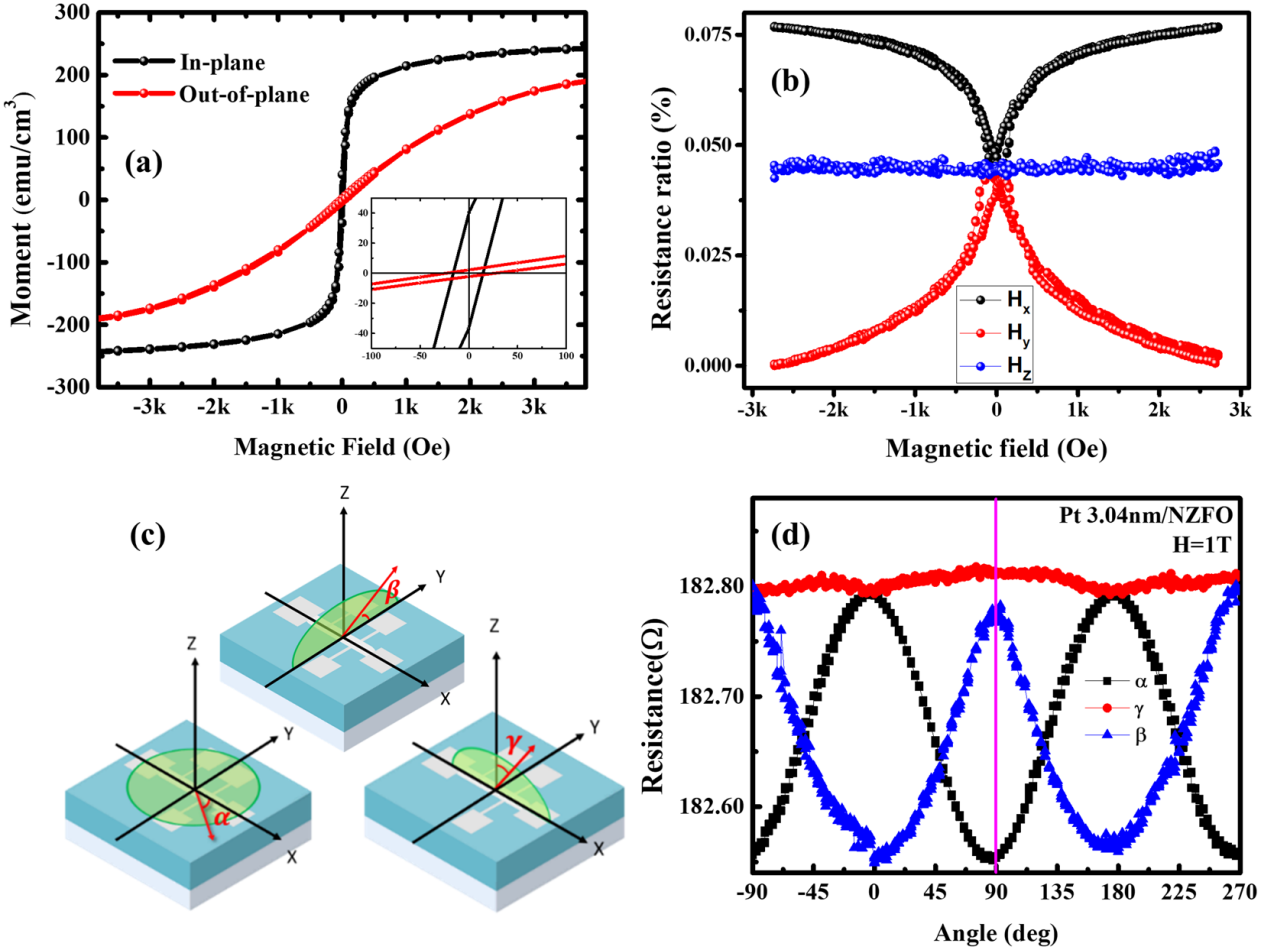
(**a**) Hysteresis loops of single NZFO layer with out-of-plane and in-plane magnetic fields. (**b**) Field-dependent MR of Pt/NZFO bilayer structure with x, y and z magnetic field directions. (**c**) Schematic diagrams of angular-dependent MR measurement with three different rotation angle α, β and γ. The charge currents are derived at the x direction for all rotation geometry. (**d**) The angular-dependent MR of Pt (d = 3.04 nm)/NZFO bilayer structure with α, β and γ rotation planes.

After structure analysis, the magnetic-field-dependent longitudinal resistance (R_xx_) of Pt/NZFO bilayer sample with 3-nm Pt thickness was measured, as shown in Fig. 3(b). The result showed that the bilayer samples had an MR-like behavior at H_x_ and H_y_ direction. Compared to NZFO in-plane hysteresis loop, as shown in Fig. 3(a), the coercive field of NZFO also matched with the observed MR. This suggests that the MR signal of the bilayer samples resulted from the interaction between NZFO and Pt. On the other hand, angular-dependent MR measurement can help identify the MR mechanism that results from SMR or anisotropic magnetoresistance (AMR). Therefore, in the Pt/NZFO bilayer structure, we elaborated three different rotation angles of longitudinal resistance, as shown in Fig. 3(d). The schematic diagrams of the different rotation angles, α, β, and γ, are also illustrated in Fig. 3(c), representing rotation in the x-y, y-z and x-z plane, respectively. The difference between the SMR and the AMR behaviors was revealed in angular-dependent MR results. For the AMR behavior, the resistance depends on the angle between the charge current and the magnetization direction (${\rho }_{xx}={\rho }_{0}+\Delta {\rho }_{A}{m}_{x}^{2}$), which is completely different from SMR (${\rho }_{xx}={\rho }_{0}-\Delta {\rho }_{s}{m}_{y}^{2}$). The MR curves of angles α and β were functions of $co{s}^{2}\theta$, while the angle γ was nearly constant. In addition, the marked purple line intersected with three MR curves, representing R_x_, R_z_, and R_y_ for different rotation angles α, γ, and β, respectively, where R_i_ (i = x, y and z) is the resistance measured with external magnetic fields. A clear SMR behavior was observed (R_x_ ≈ R_z_ > R_y_), which differed from the AMR behavior (R_x_ > R_z_ ≈ R_y_)^21,24^. As a result, the angular-dependent MR results revealed that the SMR behavior clearly dominated in the Pt/NZFO bilayer structure.

**Figure 4:**
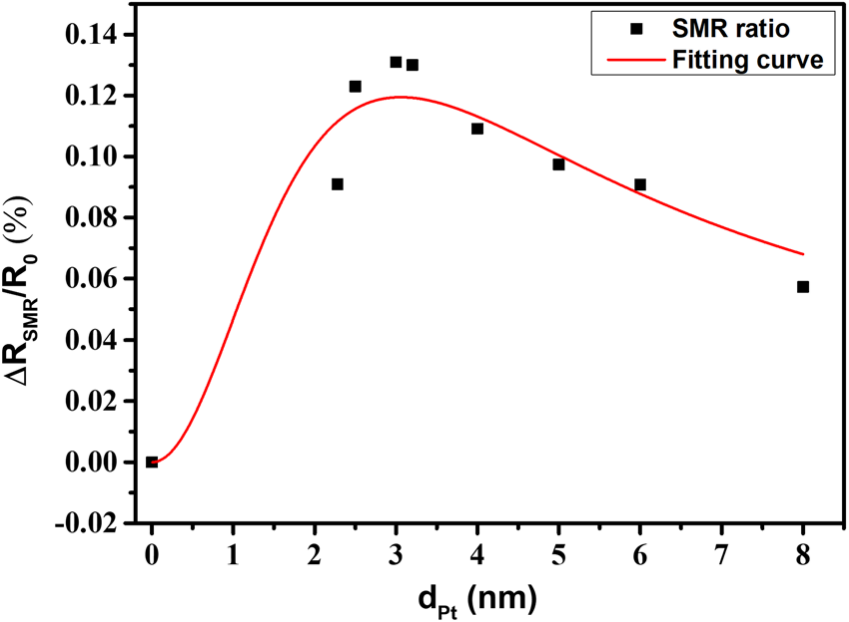
SMR ratios of Pt/NZFO bilayer structure with different Pt thickness. *R*_0_ is base resistivity which is measured at zero magnetic field, and Δ*R*_*SMR*_ is the average of the wave crest minus the average of wave trough in beta angle (${\rm{\Delta }}{R}_{SMR}={R}_{max}-{R}_{min}$). The red line represents the fitting curve by longitudinal SMR function (eq. 1).

After that, the SMR change ratios of the Pt/NZFO bilayer structure were plotted as a function of Pt thickness from 0 nm to 6 nm, as shown in Fig. 4. Moreover, the red line in Fig. 4 is the fitting curve obtained by the longitudinal SMR function^21,22^ as

1 $$\frac{{\rm{\Delta }}\rho s}{\rho }={\theta }_{SH}^{2}\frac{\lambda }{{d}_{N}}\frac{2\lambda {G}_{r}\,\tan \,{{h}}^{2}\frac{{d}_{N}}{2\lambda }}{\sigma +2\lambda {G}_{r}\,\cot \,{\rm{h}}\frac{{d}_{N}}{\lambda }},$$

where *ρ*,*σ*,*λ* and *d*_*N*_ represent the resistivity, conductivity, spin diffusion length, and thickness of the HM layer, respectively. G_r_ is interface spin-mixing conductance, which is about 1.2×10^18^ *m* ^−2^ by ferromagnetic resonance (FMR). Moreover, *θ*_*SH*_ (spin Hall angle) and λ_*pt*_ (spin diffusion length) are the fitting parameters. The theoretical fitting results well matched the experimental data points, and the fitting result provided values for *θ*_*SH*_ and λ_*pt*_ as 0.0648 and 1.31 nm, respectively. Compared with literature, our calculations for *θ*_*SH*_ and λ_*pt*_ are in reasonable ranges. Particularly, the MR ratio of the bilayer structure (Pt = 3.04 nm /NZFO) had the highest changing ratio of about 0.135% and much higher than the Pt/YIG system in literature^27,28,36,37^, which is due to significant saturation magnetization of NZFO.

**Figure 5:**
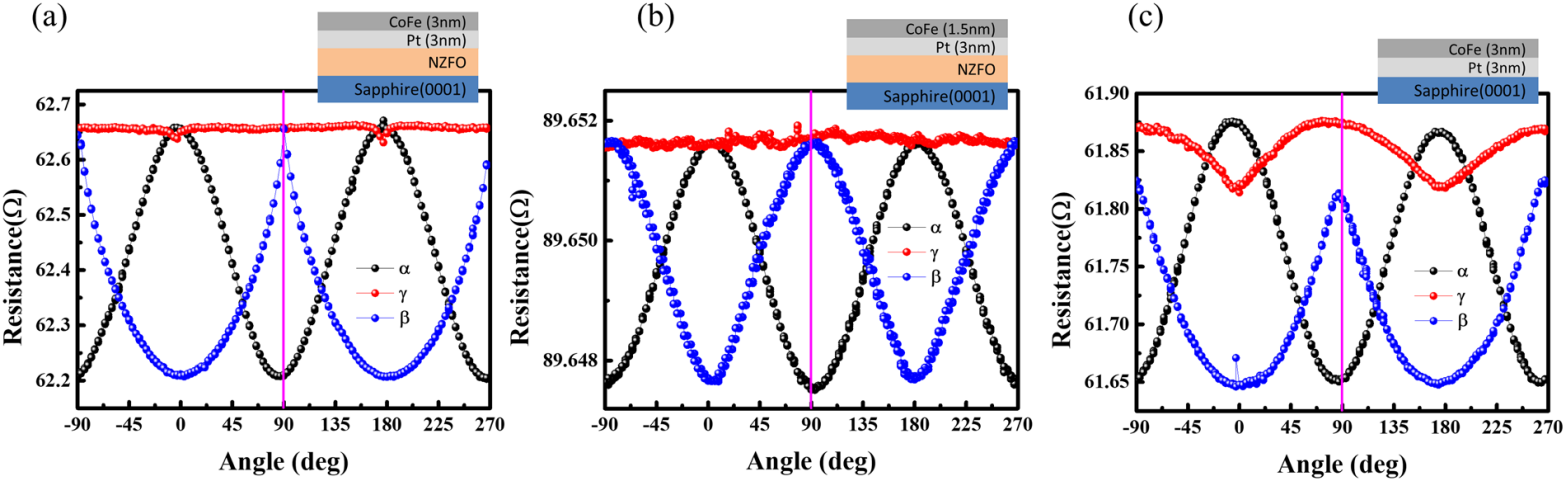
Angular-dependent MR ratio of (**a**) (**b**) tri-layer CoFe/Pt/NZFO structure for 3 nm and 1.5 nm CoFe thickness, and (**c**) bilayer CoFe/Pt structure. The measurement magnetic field is 1 T for all angular-dependent MR results.

In the above studies, the Pt/NZFO bilayer structure samples exhibited typical SMR behavior with high performance change ratio. However, increasing the interfaces number is beneficial for increasing the SMR ratio. Therefore, we present an amorphous magnetic metal layer CoFe to cover our bilayer samples, thus forming a CoFe/Pt/NZFO tri-layer structure. The highest SMR ratio of Pt(3.04 nm)/NZFO bilayer was chosen for the fabrication of tri-layer device. The angular-dependent MR of CoFe/Pt/NZFO and CoFe/Pt is shown in Fig. 5(a) and (c) respectively. The results demonstrate that the SMR behavior dominates in the tri-layer structure, which is also confirmed by the intersection of the purple line (R_x_ ≈ R_z_ > R_y_). Interestingly, in the tri-layer structure, the SMR ratio (around 0.72%) was much higher than the ratios of Pt(3 nm)/NZFO and CoFe(3 nm)/Pt(3 nm), which were only 0.135% and 0.259%, respectively. The result indicate that the tri-layer structure can significantly magnify the SMR effect, and also imply that the additional spin current may be injected into Pt layer.

**Figure 6:**
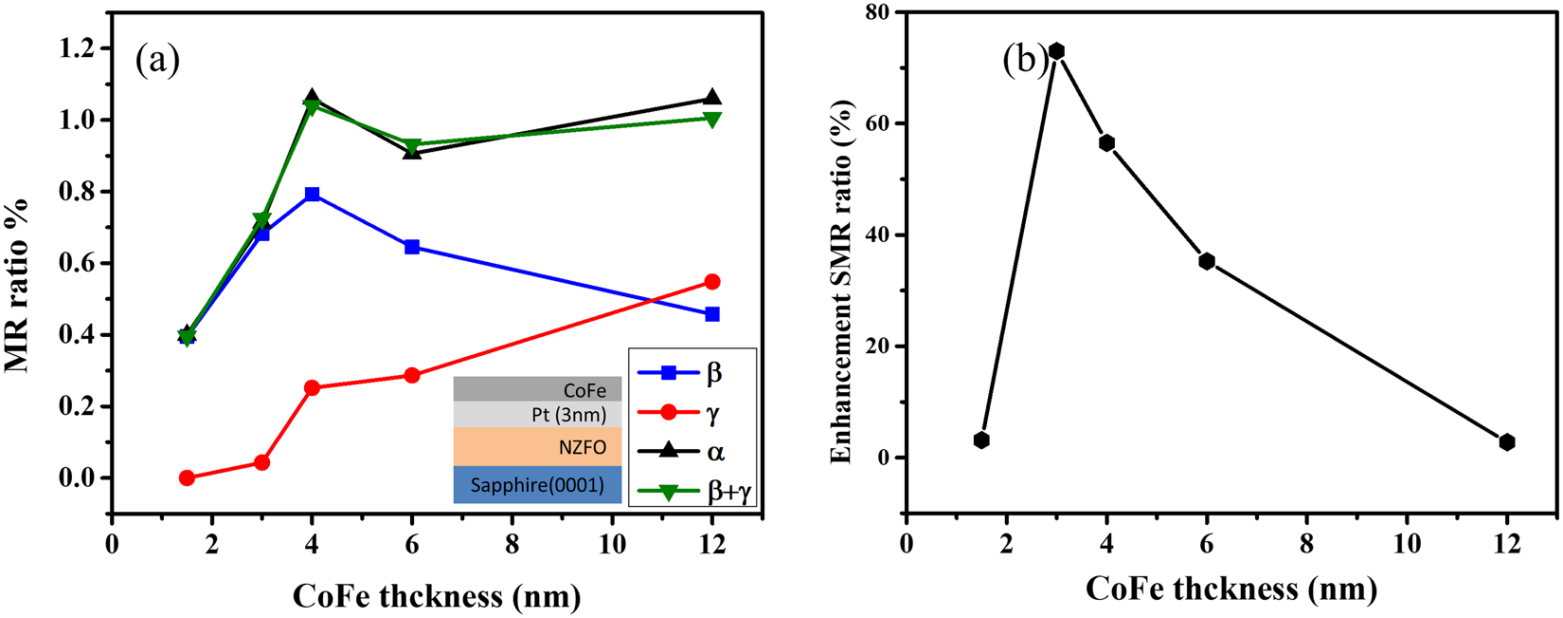
CoFe thickness-dependent MR ratios with different rotation angles α, β, γ and β+γ.

Furthermore, to realize MR behaviors of the tri-layer structure, we prepared a group of tri-layer samples with varying CoFe layer thicknesses. CoFe thickness vs. angular-dependent MR ratios with different rotation angles, α, β, γ, and ${\rm{\beta }}\,+\,{\rm{\gamma }}$, is shown in Fig. 6. First, we observed that the MR ratios of α angle (black triangle) was very similar to the ratios of β+γ angle (green triangle), which represents the satisfied rotation experimental result. Furthermore, the MR ratios of β (blue square) and γ (red circle) were dominated by the SMR and AMR behavior, respectively, as per the abovementioned results of the bilayer structure. Particularly, the results of the γ angle with varying CoFe thickness showed that no AMR behavior arises when the CoFe thickness is below 3 nm; conversely, the AMR behavior arises when the CoFe thickness is above 3 nm. Comparing with literature of the FM/HM bilayer structure^34^, we suggest that the results of γ angle are due to the proportion of CoFe (9.9×10^−5^ cm) and Pt (3.2×10^−5^ cm) resistivity, where the driving current mostly flows in Pt when the CoFe thickness is below 3 nm. Moreover, the MR ratios of β angle with varying CoFe thickness increase at first, but then decrease with the increase of the CoFe thickness, until a maximum MR ratio is observed at 4 nm CoFe thickness. We suggest the following scenario: when the resistances of CoFe and Pt are significantly different, the electrons are mainly moving with simplified path, which means that the driving current prefer to transmit in low resistance layer, focusing on Pt. In contrast, when the resistances of CoFe and Pt layers are similar, the interface-induced multi-reflective electron should be generated^38^, leading to the injection of the additional spin current in the Pt layer. Therefore, the SMR behavior should arise at an intermediate CoFe thickness. Finally, we summarize the three types of structures (CoFe/PT/NZFO, CoFe/PT and PT/NZFO) of SMR changing ratio (β angle) for different CoFe layer thicknesses, as shown in Fig. 6(b), in which the y-axis is defined as the enhanced SMR ratio $(\frac{SMR(CoFe/Pt/NZFO)-(SMR(Pt/NZFO)+SMR(CoFe/Pt))}{SMR(Pt/NZFO)+SMR(CoFe/Pt)} \% )$. Spuriously, the result shows that the MR ratio can be enhanced and the highest enhancement SMR ratio is obtained at 3 nm CoFe thickness, which can be enhanced by over 70%. The tendency of enhancement SMR ratio may be correlated with our discussion of the current separation in CoFe and Pt. However, the mechanism of SMR ratio enhancement and the spin injection in tri-layer structure still remains unclear. Therefore, a further investigation of microscopic spin current motion in interface is needed to deeply understand the SMR mechanism. The SMR behavior is a fascinating concept in spintronics and offers exciting insights in related spin transport behavior. Our finding in CoFe/Pt/NZFO tri-layer structure not only significantly enlarge the SMR signal but also systemically study the SMR behavior and related phenomena in multi-layer structure.

## Conclusion

We successfully grew three different arrangement samples, Pt/NZFO, CoFe/Pt and CoFe/Pt/NZFO by the PLD system, demonstrating that those films are high quality samples, as confirmed by RHEED, XRD, and AFM. Therefore, we quantitatively investigated the SMR effect in Pt/NZFO, CoFe/Pt and CoFe/Pt/NZFO heterostructures. In the Pt/NZFO bilayer system, we clearly observed a maximum SMR in our samples with a ratio of 0.135% at a Pt thickness of around 3 nm, and we obtained *θ*_*sh*_, λ_*pt*_ and G_r_ by fitting the longitudinal SMR function. In addition, we also showed that the AMR behavior can be ruled out in this study. Moreover, the tri-layer structure of CoFe/Pt/NZFO efficiently enhanced the SMR ratio, which is much larger than the bilayer system (Both Pt/NZFO and CoFe/Pt), observing by ADMR measurement.

### Experimental Procedures

In this experiment, two sets of heterostructure films, as shown in Fig. 2a (bilayer of Pt/NZFO/Sub. and tri-layer of CoFe/Pt/NZFO/Sub.), were *in situ* deposited on (0001) C-sapphire substrates by the pulse laser deposition (PLD) technique. The Hall bar shadow mask was used *in situ* to pattern the samples for transport measurement (the ratio of length and width being 1). The NZFO films were grown at 750 °C with laser energy 2 J/cm^2^. Pt and CoFe were grown at room temperature with different laser energies of 4 J/cm^2^ and 3 J/cm^2^, respectively. After deposition, reflection high-energy electron diffraction (RHEED) *in situ* monitored the quality of the films, and the structural properties were investigated by X-ray diffraction (XRD) and atomic force microscopy (AFM). The magnetic properties of NZFO were measured by a superconducting quantum interference device. The angular-dependent MR was measured by our lab-made rotation system and the DC voltage signal was detected by a nano-voltage meter, Keithley AC 2182 A, at room temperature, and all MR ratios are defined as (R_max_-R_min_)/R_min_ in this paper.
